# Mouse Model of Sutured Endothelial Keratoplasty Technique

**DOI:** 10.3390/jcm14134442

**Published:** 2025-06-23

**Authors:** Junki Kurita, Takahiko Hayashi, Chihiro Sunouchi, Toshiki Shimizu, Yusuke Hara, Noriko Inada, Jun Shoji, Satoru Yamagami

**Affiliations:** 1 Division of Ophthalmology, Department of Visual Sciences, Nihon University School of Medicine, Tokyo 173-8610, Japan; takamed@gmail.com (T.H.); yamagami.satoru@nihon-u.ac.jp (S.Y.); 2 Department of Ophthalmology, Higashimatsuyama Municipal Hospital, Saitama 355-0005, Japan

**Keywords:** mouse model of corneal transplantation, penetrating keratoplasty, endothelial keratoplasty

## Abstract

**Background/Objectives**: In this study, a mouse model of sutured endothelial keratoplasty was established and compared with a traditional penetrating keratoplasty (PKP) model in both syngeneic (BALB/c) and allogeneic (C57/BL6) patterns. **Methods**: For the endothelial keratoplasty (EK) model, chimeric donor tissues consisting of BALB/c epithelium-stroma combined with either syngeneic (BALB/c) or allogeneic (C57/BL6) stroma-endothelium were transplanted into BALB/c mice. Graft transparency, gene expression, and mRNA levels in the transplanted tissues were assessed using polymerase chain reaction (PCR) and quantitative real-time reverse transcription PCR (qRT-PCR) to evaluate inflammatory status. **Results**: Allogeneic PKP had a higher opacity score than syngeneic PKP. In contrast, syngeneic EK mice had similar opacity scores to those of allogeneic EK mice. Upregulation of CXCR3, the receptor for CXCL10, was demonstrated by qRT-PCR in allogeneic PKP mice but not in allogeneic EK mice. **Conclusions**: Comparison between the syngeneic and allogeneic PKP groups revealed differences in CXCR3 mRNA expression, suggesting that CXCR3 could be a potential biomarker for rejection in the PKP mouse model. Additionally, the EK model did not show CXCR3 upregulation despite the opaque cornea due to nonspecific inflammation. Therefore, our mouse model was considered to be a successfully established EK model.

## 1. Introduction

Corneal transplantation is a surgical procedure used to restore vision in cases of corneal opacity [1,2]. Although corneal transplantation has a lower incidence of rejection than other organ transplants [3], post-transplant rejection remains one of the most sight-threatening complications. Therefore, managing graft rejection is crucial for maintaining corneal transparency after transplantation.

Recently, partial corneal transplantation has become increasingly popular [4,5,6]. For conditions like bullous keratopathy, where the corneal endothelium is damaged, the standard treatment involves endothelial keratoplasty (EK), such as Descemet stripping automated endothelial keratoplasty (DSAEK) and Descemet membrane endothelial keratoplasty (DMEK). Generally, DMEK and DSAEK are reported to be less prone to rejection than penetrating keratoplasty (PKP), and the rejection reactions are milder compared with those of PKP. The reasons for the milder reaction observed during graft rejection after DSAEK remain unknown [7,8,9,10].

Several mechanisms of post-transplant rejection have been reported in mouse models [11,12]. However, mice have smaller eyes than other animals, such as rabbits, making it difficult to create experimental models using these animals; however, genetic control has been established, and mice have recently been used in experiments on immune responses. Evaluations of rejection in mouse models are often based on clinical observations using microscopy; they lack objectivity and are not quantitative. These limitations led us to search for biomarkers of rejection using corneal grafts obtained postoperatively at the onset of postoperative corneal rejection. Moreover, several reasons make the creation of EK mouse models difficult, most notably the inability to obtain a postoperative resting position. The purposes of this study were to establish a new EK mouse model that overcomes that problem and to evaluate postoperative responses.

## 2. Materials and Methods

### 2.1. Animals and Anesthesia

All experiments involving mice were approved by the Animal Experiment Steering Committee of Nihon University (Approval No.: AP17M012). The animals were treated in accordance with the guidelines of the Association for Research in Vision and Ophthalmology Statement for the Use of Animals in Ophthalmic and Vision Research and the Public Health Policy on Humane Care and Use of Laboratory Animals (U.S. Public Health Service) and in accordance with the ARRIVE guidelines. BALB/c and C57BL/6 mice, purchased from Charles River Japan (Yokohama, Japan), were used to generate two mouse models of keratoplasty (PKP and EK). All male mice, aged 6 to 12 weeks, were housed in a pathogen-free environment. All procedures, including corneal transplantation, were performed under general anesthesia using 0.1 mL of a triad of intraperitoneal anesthetics (medetomidine (1 mg/mL) (ZENOAQ, Fukushima, Japan), midazolam (5 mg/mL) (Sandoz, Tokyo, Japan), and butorphanol tartrate (5 mg/mL) (Meiji, Tokyo, Japan)). In total, 96 mice were randomly assigned to 24 cages and used for our two-group experiments (n = 4 mice per cage). The group allocation was managed by SY.

### 2.2. Corneal Transplantation

#### 2.2.1. A Mouse Model of PKP

**Figure 1:**
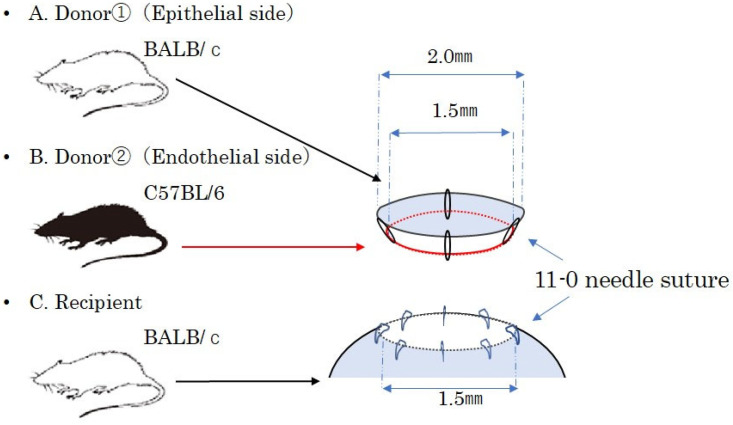
A schematic representation of reconstituted chimeric allografts. (**A**) The donor, consisting of the anterior part (epithelial and stroma from BALB/c) and (**B**) the posterior part (stroma and endothelium from C57BL/6), was transplanted to recipients. (**C**) The diameter of the donor graft was 2.0 mm, and the recipient incision wound was 1.5 mm.

BALB/c mice were used as recipients, with age-matched BALB/c mice serving as donors for the syngeneic PKP model and age-matched C57BL/6 mice for the allogeneic PKP model. The right eye of the recipient mouse was marked with a 1.5 mm trephine blade, and the cornea was excised along the markings using Vannas scissors. The cornea of the donor mouse was marked with a 2.0 mm trephine blade and excised with Vannas scissors to create the corneal graft. The corneal graft was sutured in eight locations with 11-0 (0.1 metric) needle sutures (MANI^®^ Ophthalmic Suture, MANI, Tochigi, Japan) onto the right eye of the recipient mouse. After the procedure, the ocular surface was treated with ofloxacin ophthalmic ointment (Tarivid^®^ Ophthalmic Ointment, Santen, Osaka, Japan), and the eyelid was sutured closed with 6-0 needle sutures (Ethilon^®^ Nylon Suture, Johnson & Johnson, New Brunswick, NJ, USA) (Figure 1). The eyelid and corneal sutures were removed 1 week after surgery. Four weeks after surgery, 1 mL of a triad of anesthetics was administered intraperitoneally for general anesthesia, and the anterior segment of the eye was observed and photographed under a stereomicroscope. After assessing the degree of rejection, the eyeball was removed under general anesthesia, and the mice were euthanized by cervical dislocation. Eyes with postoperative cataracts, infections, or anterior chamber loss were excluded from the study.

#### 2.2.2. Creation of a Mouse EK Model

EK typically relies on air pressure in the anterior chamber to attach the graft, followed by a resting position to ensure graft adherence. However, maintaining a postoperative resting position in mice is challenging. In this study, we developed a new mouse EK model that uses sutures instead of air pressure to secure the graft.

An allogeneic EK model was created using BALB/c and C57BL/6 mice, with corneal transplantation performed under general anesthesia, similar to the PKP mouse model. The corneas were excised using Vanna scissors. The whole cornea was obtained and then separated into the anterior layer from BALB/c mice and the posterior layer from C57BL/6 mice using Vannas scissors. The whole cornea was grasped with forceps and divided into two layers: an epithelial-side graft with stroma and an endothelial-side graft with stroma. A composite corneal graft was prepared by suturing four locations using 11-0 (0.1 metric) needle sutures. The cornea of the recipient BALB/c mouse’s right eye was marked with a 1.5 mm trephine and excised using Vannas scissors. The composite corneal graft was then fixed by placing eight sutures with 11-0 (0.1 metric) sutures into the right eye of the recipient mouse. This chimeric model, composed of alloendothelium and posterior stroma after transplantation, immunologically mimics DSAEK in humans. After transplantation, the ocular surface was coated with ofloxacin ophthalmic ointment (Tarivid^®^ ophthalmic ointment), and the eyelid was sutured closed with a 5-0 (1.0 metric) needle suture (MANI^®^ Ophthalmic Suture, MANI, Tochigi, Japan) (Figure 1). The syngeneic EK model was created using the same technique, substituting BALB/c mice for C57BL/6. One week after surgery, the eyelid and corneal sutures were removed. The anterior segment of the eye was observed and photographed using a stereomicroscope. After assessing the degree of rejection, the operated eyes and draining lymph nodes were removed under general anesthesia. Eyes with postoperative cataracts, infections, or anterior chamber loss were excluded from the study.

### 2.3. Evaluation and Determination of Rejection After Corneal Transplantation

In the PKP model, the transplanted cornea was evaluated 4 weeks after the procedure by scoring the transplanted eye using a stereomicroscope. The scoring was performed by two experienced physicians, following evaluation methods previously reported by J. Plšková and Guo-Ling Chen et al. [13,14]. The degree of vascular invasion of the corneal graft was assessed based on the extent of progression from the periphery to the center of the graft, using a 5-point scale ranging from 0 to 4. The opacity of the corneal graft was scored as follows: 0 points: Clear corneal graft; 1 point: minimal, superficial opacity not involving the stroma; the pupil margin and iris texture are readily visible; 2 points: minimal to moderate opacity involving the corneal stroma; the pupil margin and iris texture are visible; 3 points: moderate stromal opacity; only the pupil margin is visible; 4 points: intense stromal opacity; only the outline of the pupil is visible; and 5 points: severe stromal opacity; the anterior chamber is invisible. The degree of vascular invasion was scored as follows: 0 points: no neovascularization; 1 point: neovascularization around the recipient graft beds only; 2 points: neovascularization around the peripheral graft; 3 points: the middle and peripheral parts of the graft are vascularized; and 4 points: the entire cornea is vascularized. Corneal rejection in the EK model was evaluated using the same criteria as described for the PKP model.

### 2.4. Laser Microdissection Method

To study immune response-related gene expression, corneal tissue was harvested from unfixed frozen sections using laser microdissection. Four weeks after the procedure, the extracted eye and eyelid were left unfixed and embedded in an OCT compound. Eight-micrometer frozen sections were then prepared using a cryostat (CM3050S, Leica, Wetzlar, Germany). The frozen sections were fixed in cold 100% methanol for 30 s and stained with 0.02% toluidine blue. The grafted corneas were then excised from the frozen sections using laser microdissection (LMD6/7, Leica, Germany). All layers of the cornea, from the epithelial to the endothelial layer, were sectioned to include only the graft and collected from a total of 18 frozen sections. The excised corneas were collected in Thermo-Tube caps (Thermo Fisher Scientific, Waltham, MA, USA) containing 10 µL of mineral oil (Thermo Fisher Scientific) and prepared as samples. Messenger RNA (mRNA) was extracted from these samples using a nucleic acid extraction kit (MagDEA Dx SV RNA, Precision System Science, Chiba, Japan) and converted to cDNA using a high-capacity cDNA reverse transcription kit (Life Technologies Japan, Tokyo, Japan). The cDNA was then stored at −80 °C until further use.

### 2.5. PCR Array

A polymerase chain reaction (PCR) array was used to screen for immune response-related genes expressed in the corneal grafts of the PKP model. Samples from the allogeneic PKP model obtained via laser microdissection were analyzed using the PCR array. Healthy corneal samples, obtained using the same technique from untreated mice of the same age, served as controls. Following mRNA extraction, cDNA was prepared using the RT2 First Strand Kit (QIAGEN, Hilden, Germany).

The PCR array method was used for the simultaneous analysis of multiple gene expressions using RT2 Profiler PCR Arrays (QIAGEN). The RT2 Profiler PCR Array for Mouse Innate & Adaptive Immune Responses (PAMM-052ZA, QIAGEN) was used for this experiment. The reaction solution was prepared according to the manufacturer’s instructions and analyzed using a real-time cycler (StepOnePlusTM, Thermo Fisher Scientific). The results were processed with ansan RT2 Profiler, and fold changes were calculated and converted to ΔΔCt values using RT2 Profiler PCR Array software [15].

### 2.6. Quantitative Polymerase Chain Reaction (qRT-PCR)

qRT-PCR was used to evaluate differences in the expression of immune response-related genes between PKP models and controls based on the PCR array results. The qRT-PCR assay was performed using samples from the syngeneic PKP models (n = 5), allogeneic PKP models (n = 5), syngeneic EK models (n = 5), and allogeneic EK models (n = 5) for genes that showed differences between the PKP mouse model and the controls in the PCR array. TaqMan Gene Expression Assays (Thermo Fisher Scientific) were used as primers and probes for the qRT-PCR assay. The assay items were selected for their high specificity, given the quantitative nature of the corneal graft samples. The genes analyzed included CXCL10 (Mm00445235_m1), its receptors CXCR3 (Mm999999054_s1), CCL5 (Mm01302427_m1), and CCR5 (Mm01962351_s1). Real-time RT-PCR was conducted using a thermal cycler (QuantStudio 3, Thermo Fisher Scientific). Results were calculated using the ΔΔCt method with β-Actin (Mm0607939_s1) as the endogenous control [15].

### 2.7. Statistical Analyses

SPSS version 25 (IBM, NY, USA) and EZR version 1.36 (Saitama Medical Center, Jichi Medical University, Saitama, Japan) were used for the statistical analyses. Fisher’s exact probability test was employed to compare the clinical scores. The qPCR results were analyzed using the Mann–Whitney U test for comparisons between two groups, with a one-way analysis of variance (one-way ANOVA) and Tukey’s honestly significant difference test used for comparisons among four groups. The sample size was based on a previous study [16].

## 3. Results

### 3.1. Mouse PKP Model

#### 3.1.1. Corneal Opacity Score and Rates of Graft Rejection

In the syngeneic group (n = 10), the corneal opacity score was 1.1 ± 0.7 (mean ± standard deviation), and all 10 mice had a score of 2 or lower, indicating a 0% incidence of graft rejection. In contrast, the allogeneic group (n = 10) had a corneal opacity score of 2.9 ± 0.8, with vascular invasion observed in all 10 mice. Among these, four mice had a score of 2 or lower, while six mice had a score of 3 or higher, resulting in a 60% incidence of graft rejection. A statistically significant difference was found in the average corneal opacity score between the two groups (*p* < 0.011) (Table 1).

#### 3.1.2. Detection of Biomarkers of Post-Transplant Corneal Rejection

Screening of candidate biomarker genes using PCR array: A PCR array was utilized to screen for potential rejection biomarkers. Normal eye samples were used as controls to identify critical factors that were upregulated in allogeneic mouse models (n = 6). The candidate biomarkers selected based on this screening included CD14, CXCL10 (IP-10), and CCL5 (RANTES).

**Figure 2:**
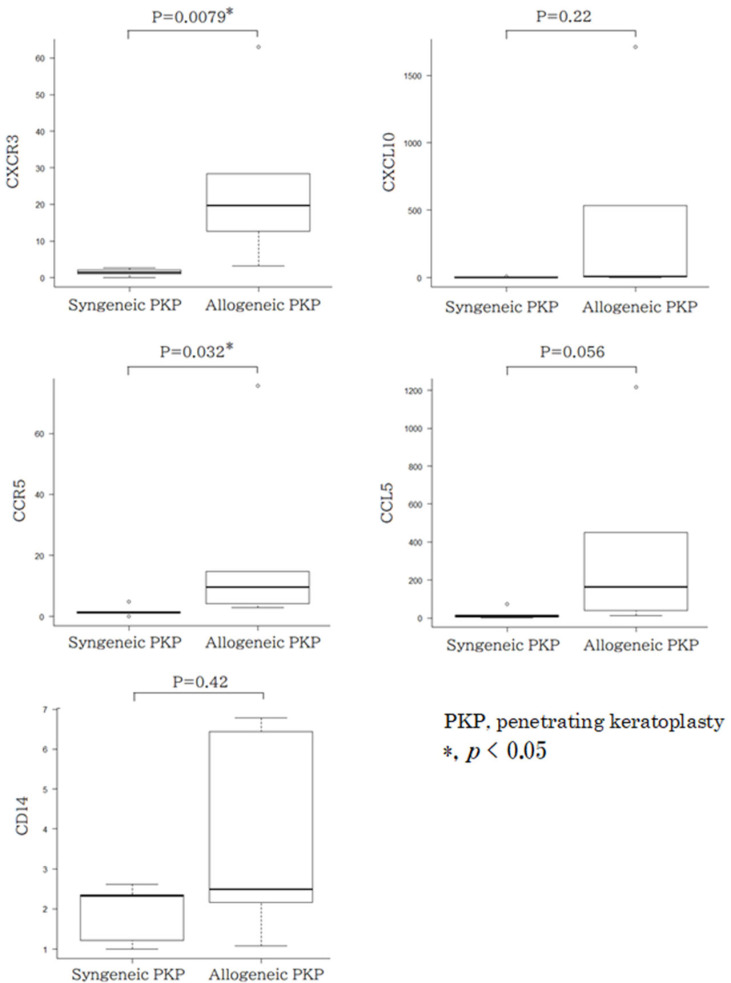
Determination of gene expression levels with qRT-PCR. Significant differences in CXCR3 and CCR5 mRNA expression levels were obtained between the allograft and allogeneic groups (CXCR3 *p* = 0.079, CCR5 *p* = 0.032). On the other hand, no statistically significant differences were observed in CXCL10/IP-10 (*p* = 0.22), CCL5/RANTES (*p* = 0.056), or CD14 (*p* = 0.42) mRNA expression levels.

Determination of biomarkers by comparing syngeneic and allogeneic groups: To identify critical biomarkers, qRT-PCR was performed based on the results of the PCR array results. Five eyes were selected from each group for analysis. In the syngeneic group, all eyes had scores of 2 or lower (0 eyes had a score of 3 or higher). In the allogeneic group, four eyes had scores of 3 or higher, while one eye had a score of 2 or lower. Due to the limited availability of samples, CD14, CXCL10-CXCR3, and CCL5-CCR5 systems were selected as target genes. CXCR3 mRNA expression was 1.4 (median) (range: 1.0–2.7) in the syngeneic group and 20 (median) (range: 3.1–63) in the allogeneic group, with the allogeneic group showing significantly higher expression than the syngeneic group (*p* = 0.0079). Significant differences were also observed in CCR5 mRNA expression levels (*p* = 0.032). However, no statistically significant differences were found in the expression levels of CXCL10/IP-10 (*p* = 0.22), CCL5/RANTES (*p* = 0.056), and CD14 (*p* = 0.42) between the allogeneic and syngeneic groups (Figure 2).

### 3.2. Mouse EK Model

#### 3.2.1. Corneal Stromal Opacity Score in a Mouse EK Model

We assessed a novel mouse EK model by evaluating the corneal stromal opacity score, following the approach used for the mouse PKP model. Table 2 shows the relationship between the clinical scores and CXCR3 expression in the syngeneic EK group (n = 5) and the allogeneic EK group (n = 5). The mean corneal stromal opacity scores were 3.2 ± 0.4 (mean ± standard deviation) for the syngeneic EK group and 3.0 ± 1.3 for the allogeneic EK group, indicating no significant difference between the groups (*p* = 1). However, significant differences were observed between the syngeneic EK and syngeneic PKP groups in eyes with corneal opacity (*p* = 0.00033) and between the allogeneic EK and syngeneic PKP groups (*p* = 0.0037). Conversely, no significant differences were found between the syngeneic EK and allogeneic PKP groups (*p* = 0.23) and between the allogeneic EK and allogeneic PKP groups (*p* = 0.6) (Table 2). These findings suggest that the corneal opacity score may not be a definitive indicator for distinguishing whether a rejection reaction has occurred.

#### 3.2.2. Comparison Between the Syngeneic and Allogeneic Groups by CXCR3 Expression

**Figure 3:**
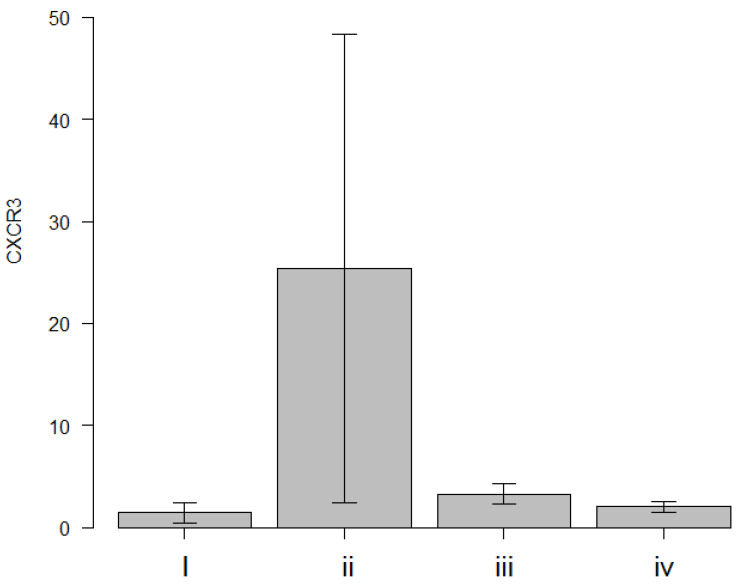
Comparison of CXCR3 mRNA expression: (i) syngeneic PKP; (ii) allogeneic PKP; (iii) syngeneic EK; (iv) allogeneic EK groups. CXCR3 mRNA expression in the allogeneic PKP group was the highest among the four groups, and all showed significant differences compared with the other groups (syngeneic PKP-allogeneic PKP, *p* = 0.022; allogeneic PKP-syngeneic EK, *p* = 0.036; allogeneic PKP-allogeneic EK, *p* = 0.026; Tukey’s honestly significant difference test). CXCR3 mRNA expression was lower in the allogeneic and syngeneic EK groups, but there was no statistically significant difference between the two groups.

We identified CXCR3 as a key biomarker for histological discrimination. qRT-PCR was performed to compare the CXCR3 mRNA expression levels in the syngeneic and allogeneic PKP and EK groups using the whole corneal samples collected 4 weeks post-surgery. Figure 3 illustrates the CXCR3 mRNA expression in each group: (i) syngeneic PKP; (ii) allogeneic PKP; (iii) syngeneic EK; (iv) allogeneic EK. First, one-way ANOVA was performed, and significant differences were found among the four groups based on a comparison with at least one group (*p* = 0.012). Then, Tukey’s honest significant difference test was performed to determine which group was associated with the significant differences. The results showed that the allogeneic PKP group exhibited the highest CXCR3 mRNA expression among all four groups (syngeneic PKP vs. allogeneic PKP, *p* = 0.022; allogeneic PKP vs. syngeneic EK, *p* = 0.036; allogeneic PKP vs. allogeneic EK, *p* = 0.026). CXCR3 mRNA expression was notably low in both the syngeneic EK and allogeneic EK groups, with no statistically significant differences detected between these groups (Figure 3).

## 4. Discussion

Currently, the immune response associated with allograft rejection after corneal transplantation remains incompletely understood despite significant progress through experiments using mouse models. In animal models of full-thickness corneal transplantation (PKP), allograft rejection is typically assessed by scoring the degree of corneal opacity and the extent of vascular invasion in the corneal grafts under a microscope [14]. While these evaluations are common, they have notable limitations in terms of objectivity. The scoring system can be significantly influenced by observer subjectivity, and the lack of quantification in the current evaluation methods impairs objectivity, reproducibility, and accuracy. Therefore, to accurately assess both the severity and presence of rejection, establishing a rejection biomarker is crucial. A biomarker would not only facilitate quantitative evaluation but also offer insights into the underlying mechanisms of rejection based on genotype.

However, in recent years, a significant number of patients requiring corneal transplantation have undergone partial-thickness transplantation. Notably, approximately half of these transplantations involve endothelial keratoplasties, such as DSAEK or DMEK. Consequently, developing a new mouse model using a partial-thickness endothelial allograft is crucial for understanding the precise mechanisms underlying corneal endothelial transplantation. Reports suggest that the incidence of graft rejection following EK is lower than that following PKP [8,17]. Several theories support the lower rejection rates associated with EK. One theory is the anterior chamber-associated immune deviation (ACAID), which suggests that alloantigens transplanted into the anterior chamber can induce ACAID, thereby protecting the donor tissue from the host’s immune system. Another theory posits that a smaller volume of transplanted tissue in EK results in fewer antigen-presenting cells and antigens, which may reduce the likelihood of graft rejection [9].

**Figure 4:**
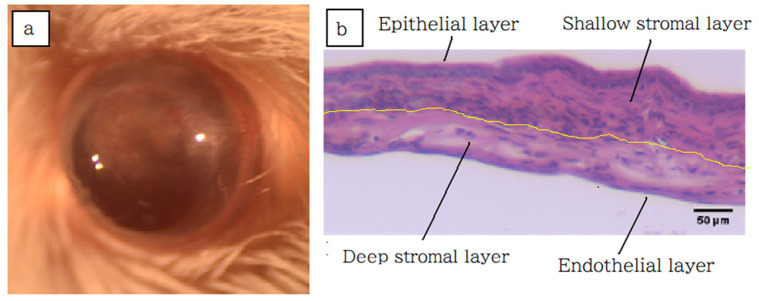
Anterior segment photo and hematoxylin and eosin (HE) staining in syngeneic EK mouse model. The syngeneic endothelial keratoplasty (EK) mouse model was enucleated 4 weeks after surgery, and the sections were stained with HE. (**a**) Anterior segment of the eye four weeks after surgery showing corneal graft opacity and neovascular invasion. (**b**) HE staining of the corneal graft. The border between the epithelial and endothelial grafts is indistinct but is thought to be a yellow line with cellular infiltration.

In this study, we utilized four corneal transplantation groups: syngeneic PKP, allogeneic PKP, syngeneic EK, and allogeneic EK mouse models. While the PKP model is well-established for studying posttransplant rejection, the EK mouse model has not been fully developed. EK procedures, such as DSAEK or DMEK, typically require the donor cornea to adhere to the recipient’s back surface with the aid of air injected into the anterior chamber, necessitating a prolonged supine rest. Maintaining this supine position in mice post-surgery presents significant challenges. We developed a mouse model for EK by using anchoring sutures to fix the transplanted graft, offering an alternative to pneumatic adhesion, similar to methods used in complex DSAEK cases. Our model employed chimeric grafts composed of a syngeneic anterior layer (epithelium and stroma) and either an allogeneic or syngeneic posterior layer (stroma and endothelium). While these chimeric grafts are not entirely equivalent to human DSAEK or DMEK procedures, they effectively simulated the conditions. Examination of the chimeric corneal grafts obtained 4 weeks post-surgery revealed the presence of a corneal endothelial layer from the deep corneal stroma, confirming the validity of the model (Figure 4). Thus, we have successfully established a stable EK-like mouse model.

Next, we assessed the postoperative status of the grafts in our EK mouse model using traditional scoring methods and compared these clinical scores with objective evaluations through qRT-PCR. Additionally, we compared the newly established syngeneic and allogeneic EK models with the syngeneic and allogeneic PKP models across various aspects, including clinical scores and rejection biomarkers.

In this study, the following results were obtained: (1) CXCR3 mRNA expression level was identified as a highly specific rejection biomarker when comparing immune-related factor mRNA expression between the syngeneic and allogeneic PKP groups; (2) CXCL10/IP-10, CCR5, and CCL5/RANTES mRNA expression levels were found to be upregulated, likely due to nonspecific inflammatory responses following corneal transplantation.

The upregulation of CXCR3 in the allogeneic PKP model aligns with previous findings [18]. CXCR3 is a G-protein-coupled receptor and chemokine receptor with ligands, including CXCL9/MIG, CXCL10/IP-10, and CXCL11/I-TAC. It is primarily expressed in immune cells, such as type 1 helper T cells (Th1) and natural killer cells, and is also present in some epithelial and endothelial cells. This expression pattern supports its role in mediating immune responses and graft rejection [19]. CXCR3 has been implicated in several pathologically relevant diseases, including multiple sclerosis [20,21], pulmonary fibrosis [20,22], type 1 diabetes [23], and acute heart transplant rejection [24]. It has also been linked to post-transplant rejection of the cornea [18]. In a study using an allogeneic PKP mouse model, rejection was mainly caused by Th1-type immune responses [25]. Additionally, Tan et al. demonstrated in an animal model that simultaneous administration of anti-CXCR3 and anti-CCR5 antibodies effectively suppressed rejection after corneal transplantation [18].

In the present study, CXCR3 mRNA expression in the grafted corneas of the allogeneic PKP mouse model with rejection was found to be higher compared with healthy corneas. This expression was significantly elevated in the allogeneic PKP group relative to the syngeneic PKP group. These findings suggest that the increase in CXCR3 mRNA expression is associated with an elevated number of CXCR3-positive Th1 cells in the rejected PKP grafts.

This suggests that CXCR3 could serve as a highly specific biomarker for rejection in a mouse model of corneal transplantation. Although CXCL10, a ligand for CXCR3, was found to be upregulated in allogeneic PKP compared with healthy corneas, a similar trend was also observed in syngeneic PKP. These results imply that CXCL10 may be involved in nonspecific inflammatory responses post-transplantation rather than being a specific marker for rejection.

The most critical finding of this study was that CXCR3 upregulation was observed exclusively in allogeneic PKP (Figure 3), suggesting that the immune response in allogeneic PKP differs from that in syngeneic transplantation or allogeneic EK. Additionally, the lack of CXCR3 upregulation following EK aligns with previous studies indicating a lower immune response in models using chimeric grafts [16,26] or allogeneic cultured endothelial cells [27].

One limitation of this study was the small number of cases. Therefore, it is necessary to include more cases in the future. In addition, we believe that additional tests, such as optical coherence tomography measurements, can be added to the evaluation of corneal grafts to make it more robust.

## 5. Conclusions

In conclusion, our results underscore the important role of CXCR3 in graft rejection following PKP, while the absence of CXCR3 upregulation in EK suggests a weaker immune response in this model. In other words, the mouse model created was considered useful as an EK model.

## Figures and Tables

**Table 1:** Corneal opacity scores.

	Syngeneic PKP (n = 10)	Allogeneic PKP (n = 10)	*p* *
Clinical score (corneal opacity) (mean ± standard)	1.1 ± 0.7	2.9 ± 0.8	
Score ≥ 3	0	6	0.011
Score < 3	10	4	

* Fisher’s exact probability test. PKP: penetrating keratoplasty.

**Table 2:** Corneal stromal opacity scores in the syngeneic and allogeneic EK groups.

	**Syngeneic EK (n = 5)**	*p* *	
		**Syngeneic PKP (n = 10)**	**Allogeneic PKP (n = 10)**
Clinical score (corneal opacity) (mean ± standard)	3.2 ± 1.4		
Score ≥ 3	5	0.00033	0.23
Score < 3	0		
	**Allogeneic EK (n = 5)**	*p* *	
		**Syngeneic PKP (n = 10)**	**Allogeneic PKP (n = 10)**
Clinical score (corneal opacity) (mean ± standard)	3.0 ± 1.3		
Score ≥ 3	4	0.0037	0.6
Score < 3	1		

* Fisher’s exact probability test. EK: endothelial keratoplasty; PKP: penetrating keratoplasty.

## Data Availability

The datasets used and/or analyzed in the current study are available from the corresponding author upon reasonable request.

## Abbreviations

The following abbreviations are used in this manuscript:

ACAID	Anterior chamber-associated immune deviation
DMEK	Descemet membrane endothelial keratoplasty
DSAEK	Descemet stripping automated endothelial keratoplasty
EK	Endothelial keratoplasty
PCR	Polymerase chain reaction
PKP	Penetrating keratoplasty
qRT-PCR	Quantitative real-time reverse transcription PCR
LD	Linear dichroism
